# [Retracted] DDAH2 alleviates myocardial fibrosis in diabetic cardiomyopathy through activation of the DDAH/ADMA/NOS/NO pathway in rats

**DOI:** 10.3892/ijmm.2026.5734

**Published:** 2026-01-19

**Authors:** Zhen-Dong Zhu, Ji-Ming Ye, Xue-Mei Fu, Xue-Chang Wang, Ji-Yun Ye, Xin-Ran Wu, Peng Hua, Yu-Qiong Liao, Wei Xuan, Jin-Lan Duan, Wei-Yuan Li, Hui Fu, Zhong-Hua Xia, Xuan Zhang

Int J Mol Med 43: 49-760, 2019; DOI: 10.3892/ijmm.2018.4034

Following the publication of this paper, it was drawn to the Editor's attention by a concerned reader that a pair of the data panels shown for the Masson staining experiments in Fig. 3A were overlapping, such that data which were intended to show the results from differently performed experiments had apparently been derived from the same original source. Upon performing an independent analysis of the data in this paper in the Editorial Office, it came to light that a pair of the panels in Fig. 4A also contained overlapping sections of data, and moreover, the data in Fig. 3A were strikingly similar to data which had appeared in a number of other articles that were written by different authors at different research institutes, several of which have been retracted, including one that had been published prior to the reciept of the above paper to *International Journal of Molecular Medicine*.

Owing to the fact that the contentious data in the above article were found to be strikingly similar to data that had already been published elsewhere, the Editor of *International Journal of Molecular Medicine* has decided that this paper should be retracted from the Journal. The authors were asked for an explanation to account for these concerns, but the Editorial Office did not receive a reply. The Editor apologizes to the readership for any inconvenience caused.

